# Editorial: Targeting Metabolism in Cancer Immunotherapy

**DOI:** 10.3389/fimmu.2018.02029

**Published:** 2018-09-11

**Authors:** Jason Roszik, Keiji Tanese, Suhendan Ekmekcioglu

**Affiliations:** ^1^Department of Melanoma Medical Oncology, The University of Texas MD Anderson Cancer Center, Houston, TX, United States; ^2^Department of Genomic Medicine, The University of Texas MD Anderson Cancer Center, Houston, TX, United States; ^3^Department of Dermatology, Keio University School of Medicine, Tokyo, Japan

**Keywords:** cancer, metabolism, immune cells, tumor microenvironment, immunotherapy

Immunotherapies are one of the most promising cancer treatment approaches, however, durable, long-term clinical benefit has occurred only in a relatively small subset of responding patients. In this Research Topic on “Targeting Metabolism in Cancer Immunotherapy,” the authors of six reviews and a commentary discuss and provide insights on the role of metabolism in immune cells and in the tumor microenvironment. Le Bourgeois et al. highlight the importance of metabolism in the functioning of immune cells including T-cells and macrophages, and also suggest potential metabolic targets. Importantly, the combination of drugs targeting metabolism together with checkpoint inhibitors present attractive therapeutic approaches to improve cancer therapy.

A suppressive metabolic tumor environment is one of the major reasons why cancer cells escape immune recognition and elimination. Human tumors maintain an immunosuppressive microenvironment which prevents effective immunotherapies. Kim et al. focus on the role of arginine metabolism, the crucial inflammatory process driving the survival of melanoma and other cancer cells inducing resistance to immunotherapy. Arginine metabolism is highly unique due to expression of enzymes involved, such as L-arginine which serves as a precursor for multiple metabolites. In this mini-review, the authors discuss the importance of cationic amino acid transporters (CAT) for L-arginine to be transported through the plasma membrane. The authors suggest that the diverse expression of CAT2B in tumors may be associated with an inflammatory environment in human melanoma cells. Moreover, they discuss recent results on nitric oxide synthases (NOSs), which are involved in arginine metabolism and lead to the subsequent activation of COX2 and other inflammatory factors and create a pro-oxidant microenvironment that supports tumor growth and suppresses anti-tumor immunity.

Another important molecular component which modulates immune response to cancer, indoleamine-2,3-dioxygenase 1 enzyme (IDO1), is discussed by (1) Based on previously published work, they point to that IDO1 is overexpressed in more than half of the tumors (2) that utilize IDO1-associated immunosuppressive mechanisms to promote their spread and survival (3). The role of IDO1 in cancer diagnostics and therapy has been an active area over a decade with the goal of preventing immunosuppression around tumor tissues and to enhance tumor eradication. One approach discussed by the authors is the combination of IFN-γ treatment with IDO1 inhibitors as a promising immunotherapy strategy that effectively improves antitumor immunity. However, they also conclude that these therapies usually terminate with failure because of immune evasion of stem cell-like cancer cells, which lead to metastasis formation, tumor recurrence and multidrug resistance (4). The authors also discuss current genome editing and exome sequencing technologies used to genetically target IDO.

The mammalian target of rapamycin (mTOR) signaling has been shown to play a role in cellular energy metabolism as well as in immune cell differentiation and effector functions. A review by Guri et al. details how mTOR activation modulates the tumor immune and metabolic microenvironment by affecting T lymphocytes, regulatory T cells (Tregs), tumor-associated macrophages (TAMs), myeloid-derived suppressor cells (MDSCs), cancer-associated fibroblasts (CAFs), and natural killer (NK) cells. Given the various roles of mTOR highlighted in this review, dissecting the specific oncogenic mechanism of mTOR is still a challenge. A commentary by (5) discusses how hypoxia-induced inhibition of mTOR also a key contributor to a reversible mitotic and growth arrest of HPV-positive tumor cells. Indeed, hypoxia, which is the main focus of another review article (Li et al.) in this Research Topic, has been shown to facilitate tumor progression and decrease the effectiveness of cytotoxic agents, and also facilitate the evasion of immunosurveillance. Based on the unique roles of potential targets that modulate immunosuppression in hypoxic tumors, the authors expect that therapeutics against those immune-modulating targets will synergize with immunotherapies and enhance anti-tumor activity of immune cells.

The importance of platelet metabolism with potential impact on immunotherapy is discussed by Kanikarla-Marie et al. in this Research Topic. Researchers believe that cancer may interrupt the normal functioning and production of platelets that become contributors to the pro-carcinogenic inflammatory milieu. Abnormalities in the number of platelets are commonly observed in various tumor types, including colorectal, ovarian, and breast cancer. In those cancer types, platelets contribute centrally to immune regulation, participate in the innate and adaptive immune functions and enrich the microenvironment for tumor cell survival. Tumor-educated platelets may have the potential for use in cancer diagnostic and screening methods. For instance, high platelet counts are associated with poor treatment outcome. The authors further propose that platelets not only increase in numbers and become activated when the cancer progresses, but they may also decrease to their normal levels and normal function after cancer therapy. These characteristics of platelets may be tested easily using routine blood tests and may also be prognostic or diagnostic markers.

Immunotherapy approaches have shown remarkable progress in the last decade, however, immunosuppression is still a challenge. The role of metabolism in immune cells and the tumor microenvironment is now well recognized, but further research is needed to define the role and impact of various metabolic processes. Identifying metabolic targets that facilitate immunosuppression is of key importance to be able to identify treatments to increase efficacy of immunotherapies.

## Author contributions

All authors listed have made a substantial, direct and intellectual contribution to the work, and approved it for publication.

### Conflict of interest statement

The authors declare that the research was conducted in the absence of any commercial or financial relationships that could be construed as a potential conflict of interest.
